# AI- and IoT-Enabled Solutions for Healthcare

**DOI:** 10.3390/s24082607

**Published:** 2024-04-19

**Authors:** Samaneh Kouchaki, Xiaorong Ding, Saeid Sanei

**Affiliations:** 1 Center for Vision, Speech, and Signal Processing (CVSSP), Department of Electrical and Electronic Engineering, University of Surrey, Guildford GU2 7XH, UK; s.kouchaki@surrey.ac.uk; 2 School of Life Science and Technology, University of Electronic Science and Technology of China, Chengdu 611731, China; xiaorong.ding@uestc.edu.cn; 3 Department of Electrical and Electronic Engineering, Imperial College London, London SW7 2AZ, UK

Patient care and management have entered a new arena, where intelligent technology can assist clinicians in both diagnosis and treatment. On the one hand, new powerful processing systems together with the rapidly increasing influence of artificial intelligence (AI) and machine learning (ML) have energised clinical tools and diagnostic systems; on the other hand, algorithms have been generated and embedded within wearable devices. A patient’s biological, physiological, pathological, and behavioural changes can be captured by smart sensors through well-designed Internet of Things (IoT) networks. These networks can further benefit from the advances in adaptive and consensus networking algorithms. New findings in bioengineering and biocomputing enable analysis and understanding of the captured patient data. In addition, through networking, unified patient screening solutions have become possible through IoT. These networks provide an opportunity to monitor people outside of the hospital setting, for example, in their homes, without any disruption to their daily activities. As the technology progresses, computationally exhaustive algorithms, such as deep learning, enable analysis of very large single- or multi-modal data . Hence, the challenge is to accelerate automatic diagnosis and monitoring and increase their accuracy by developing more powerful processing and learning algorithms.

The state of a patient during rest, walking, working, and sleeping can be well recognised if all the biomarkers of the physiological, biological, and behavioural changes of human body can be measured and processed. This sparks the need for the deployment of wearable multi-sensor and multimodal data collection systems and the associated AI technology. Hence, combining sensor technology, body sensor networks, and the necessary AI techniques become central to a complete solution for patient monitoring and healthcare.

Wearable technology including sensors, sensor networks, and their associated devices, central to IoT, have been used in a variety of applications including healthcare. The long-term, preferably noninvasive and nonintrusive, monitoring of the human body through recording multimodal biometrics and body state indicators is the goal of healthcare technology developers. For example, patients with diabetes need a simple noninvasive tool to monitor their blood sugar on an hourly basis. People experiencing epileptic seizures require the necessary instrumentation to alert them before any seizure onset. Stroke patients need their heart rate to be monitored constantly. These examples show how crucial and necessary wearable healthcare technologies and their associated AI-based monitoring systems are.

On the other hand, the development of mobile phones since the early 1990s and their rapid improvement over time, together with the availability of a large memory and wideband communication channels, enable effective IoT networks and significantly ease the ability to achieve the above objectives without hospitalising the patients for long time. This may be considered a revolution in human welfare. Therefore, the effective collection of biodata and biometrics from the human body, as well as their intelligent assessment, have a large impact on healthcare and the technology involved.

The measurable underlying information can not be always visualised by the naked eye; therefore, signal processing, ML, and AI techniques are being constantly researched and developed for a better understanding and recognition of the human body state from raw data records. In recent years, deep neural networks (DNNs) and convolutional neural networks (CNNs) have been developed and widely used in data-driven ML approaches. These techniques may be used not only for the automation of clinical diagnosis but also for the prediction of alarming and ambulatory events, via recurrent neural networks (RNNs), as well as the more accurate prescription of medicine.

Incorporation of AI into medical care leads to the so-called third generation of pervasive health applications. This recent branch of research aims to combine continuous health monitoring with other sources of medical information and knowledge. Thus, the main objective in third-generation applications is to integrate intelligent agents that implement technologies such as streaming and real-time processing, data mining, ML, and AI. Although the use of smart sensors and AI technology paves the way for personalised medicine, which is one of the objectives of future healthcare, developing compatible short- and long-range wireless communication systems is another essential factor in new IoT networks to enable fast, reliable, and secure patient data communication.

To deal with these challenges, the data analysis paradigms need to be continuously evolved by employing new methods and architectures for viable solutions to a wide range of clinical needs. Innovations in data processing, AI, and ML can facilitate faster patient monitoring, management, and treatment and convert a hospital-only treatment pathway into a cost-effective combined home–hospital or even outpatient alternative, which can improve the overall quality of healthcare and pave the way for personalised medicine. However, analysing the data collected in real time poses several challenges, as the data have significant artefacts due to transmission and recording limitations, are highly imbalanced and incomplete due to subject variability and resource limitations, and involve various modalities. Moreover, data labelling is cumbersome and often involves uncertainty.

In this Special Issue, state-of-the-art and novel AI and ML techniques using various IoT platforms have been proposed for healthcare applications by different authors. This Issue addresses major advances in the intelligent processing of data from wearable, portable, or implantable devices, mostly connected by IoT networks. The Issue covers a wide range of applications including physiological, pathological, gait monitoring, and pandemic-related problems and their diverse solutions. This Issue attracted many submissions, of which the following articles were published.

In Article 1, the authors developed benchmark glucose prediction models with long short-term memory RNN (LSTM) using time-series data collected from the GDm-Health platform, compared the prediction accuracy with models in the literature, and suggested an optimised clinical review schedule with the potential to reduce the overall number of blood tests for mothers with stable and within-range glucose measurements. They also concluded that the stacked LSTM model is a promising approach for capturing the patterns in such time-series data and that using a deep learning model with routine fingerstick glucose collection is a promising, predictable, and low-cost solution for blood glucose monitoring for women with gestational diabetes.

A new approach to tetanus monitoring from low-cost wearable sensors combined with a deep-learning-based automatic severity detection was proposed in Article 2. This approach can automatically triage tetanus patients and reduce the burden on hospital staff. The authors proposed a two-dimensional convolutional neural network (CNN) with a channel-wise attention mechanism for the binary classification of ECG signals. They demonstrated a good performance for the proposed methodology.

The machine learning-based solution presented in Article 3 was used to automate the Chicago classification algorithm for oesophageal motility disease identification. For their suggested deep learning model, InceptionV3 was used to identify the precise class of the integrated relaxation pressure, and DenseNet-201 CNN was used to classify the images into five different classes of swallowing disorders. With this hybrid solution, they achieved a desirable accuracy for automating the overall pipeline.

The machine learning-based stratification system proposed in Article 4 identified patients at risk of exhibiting high blood glucose levels, based on daily blood glucose measurements and electronic health record (EHR) data from patients with gestational diabetes mellitus. They trained linear and nonlinear tree-based regression models to predict the proportion of high readings (readings above the UK’s National Institute for Health and Care Excellence [NICE] guidelines) that a patient may exhibit in the upcoming days; XGBoost achieved the highest performance.

An ensemble learning approach to develop prediction models for effective detection of COVID-19 using routine laboratory blood test results was proposed in Article 5. The authors used custom CNN models as the first-stage classifier and several supervised machine learning algorithms as the second-stage classifier and concluded that an ensemble learning model based on deep neural networks (DNN), ExtraTrees, and AdaBoost provided the best results when using the San Raffaele Hospital dataset.

A novel and cost-effective solution, called thermopile-based respiratory gating, was proposed by the authors in Article 6 to provide a contactless screening system that measures the respiratory rate at a building’s entrance gate. Based on a customised thermopile array system, different image and signal processing methods were suggested to measure the respiratory rate from low-resolution thermal videos, where an automatic region-of-interest selection-based approach obtained a low error in measuring the breaths per minute rate. The authors argue that the technical validation provided by this study is helpful for designing and implementing a respiratory gating solution to prevent the spread of COVID-19 during the pandemic situation.

Gait has often been used as an effective metric for estimation of various physiological abnormalities such as ageing and Parkinson’s. In Article 7, a lightweight attention-based CNN model for wearable gait recognition was proposed. A four-layer lightweight CNN was first employed to extract gait features from the wearable inertial measurement unit (IMU) measured features. Then, a novel attention module based on contextual encoding information and depth-wise separable convolution was designed and integrated into the lightweight CNN to enhance the extracted gait features and simplify the complexity of the model. Finally, a Softmax classifier was used for classification to realise gait recognition.

A survey of very recent explainable AI (XAI) techniques used in healthcare and related medical imaging applications was presented in Article 8. The authors summarised and categorised the XAI types and highlighted the algorithms used to increase interpretability in medical imaging topics. They also focused on the challenging XAI problems in medical applications and provided guidelines to develop better interpretations of deep learning models using XAI concepts in medical image and text analysis. Furthermore, this survey provided future directions to guide developers and researchers toward prospective investigations on clinical topics, particularly on applications with medical imaging.

The role of IoT in clinical laboratory processes was explored in Article 9. Various IoT models and applications across laboratory processes between August 2015 and August 2022 have been included in this article. These works were classified based on availability, including preanalytical, analytical, and postanalytical. In this rigorous study, the authors identified, classified, and evaluated IoT applicability in clinical laboratory systems.

Various machine learning models used for the data acquired from wearable and non-wearable sensors and other IoT technologies for monitoring Parkinson’s disease were reviewed in Article 10, covering 112 studies. These studies proposed various methods, which were applied on different sensory data to address Parkinson’s disease-related problems. The most widely deployed sensors, the most addressed problems, and the best performing algorithms were highlighted, followed by suggestions of future research pathways.

